# Addressing challenges in the clinical applications associated with CRISPR/Cas9 technology and ethical questions to prevent its misuse

**DOI:** 10.1007/s13238-017-0477-4

**Published:** 2017-10-06

**Authors:** Xiang Jin Kang, Chiong Isabella Noelle Caparas, Boon Seng Soh, Yong Fan

**Affiliations:** 1 Key Laboratory for Major Obstetric Diseases of Guangdong Province, Key Laboratory of Reproduction and Genetics of Guangdong Higher Education Institutes, Center for Reproductive Medicine, The Third Affiliated Hospital of Guangzhou Medical University, Guangzhou, 510150 China; 2 Disease Modeling and Therapeutics Laboratory, A*STAR Institute of Molecular and Cell Biology, 61 Biopolis Drive Proteos, Singapore, 138673 Singapore

The recently developed RNA-guided clustered regularly interspaced short palindromic repeat (CRISPR)/CRISPR-associated 9 (Cas9) nuclease system has progressed to be an invaluable technology for genome manipulation in somatic cell types and germline model organisms. While the unprecedented advance in human embryo gene editing research has great potential in next-generation therapeutics, it raises various ethical concerns that need to be addressed before being translated for clinical use. Here, we discuss the current and potential applications of CRISPR/Cas9 technology and its limitations in clinical applications, as well as ethical and legal considerations in the treatment, disease prevention or disability in somatic cells or human embryo via gene editing.

The CRISPR/Cas9 system has been successfully utilised to introduce genetic modifications in a wide range of species, rendering it a powerful tool in genetic engineering. These applications are summarised in Table 1 below. Currently, this technology is applied in the treatment of genetic disorders in animals, but is advancing to be clinically used for the treatment of human diseases as well, specifically for those involving single gene mutations (Cox et al., 2015). Experiments to confirm that CRISPR/Cas9 technology can indeed modify pathogenic genes to treat inherited diseases have recently been carried out and reported. For example, three research groups demonstrated that the normally functioning dystrophin gene (*Dmd*) could be reintroduced in dystrophin-deficient mdx mice. This results in the improvement of muscle function extending from myofibers and cardiocytes, to muscle stem cells and even live animals (Barrangou and Doudna, 2016).

**Table 1:** A summary of genetic correction using CRISPR/Cas9 technology in cell therapy, agriculture, antimicrobials, and anti-viral treatment from 2013 onwards

Target gene	Animal model	References
RHO	Rat	Bakondi et al. (2016)
DMD	Mdx mice	Nelson et al. (2016); Long et al. (2014)
HBB	Tripronuclear human zygotes	Liang et al. (2015)
ASXL1	Mouse	Valetta et al. (2015)
PERV pol	Pig	Yang et al. (2015)
ALB	Pig	Peng et al. (2015)
ALS2	Maize	Svitashev et al. (2015)
CFTR	Human iPSCs	Firth et al. (2015)
CCR5	Cell lines	Li et al. (2015)
CXCR4	Primary human CD4^+^ T cells	Hou et al. (2015)
FANCC	Human fibroblasts	Osborn et al. (2015)
HBV	Mouse	Dong et al. (2015)
FAH	Mouse	Yin et al. (2014)
E6	Human cervical cells	Yu et al. (2015)
E7	Human papillomavirus (HPV) positive cell lines	Hu et al. (2014)
*E. coli* genome	*Escherichia coli*	Gomaa et al. (2014)
APH-3	*Staphylococcus aureus*	Bikard et al. (2014)
CRYGC	Mouse	Wu et al. (2013)
CFTR	Human intestinal stem cell organoids	Schwank et al. (2013)

Additionally, several other reports have proven the *in vivo* application of CRISPR treatments. For example targeted genome editing via CRISPR/Cas9 enabled the expression of the wild-type *Fah* gene and the survival and expansion of rescued hepatocytes in adult mouse liver. Disruptions in protein convertase/subtilisin/kexin type 9 (PCSK9) also result in subsequent changes in cholesterol metabolism seen in mouse hepatocytes. Taken together, these studies demonstrated the therapeutic potential of utilising CRISPR to correct human diseases, which arise from single-gene mutations.

Another application of CRISPR/Cas9 technology for the treatment or prevention of diseases includes the modification of somatic cells. This has been demonstrated in a recently-approved clinical trial whereby the cells of immune system of cancer patients were genetically edited as a form of cancer therapy. At present, there are a number of human clinical trials using CRISPR against lung, prostate, and renal cell cancers. Another important current use of genome editing is in the treatment of primary HIV infection, involving the elimination of the CCR5 co-receptor via *ex vivo* modification. Prior to the development of the CRISPR/Cas9 system, zinc-finger nuclease technology was utilised to disrupt the CCR5 co-receptor in HIV patients. This method was deemed as a promising approach for gene therapy and proceeded to evaluation for use in clinical trials (Tebas et al., 2014).

Recently, we and two other research groups have demonstrated that CRISPR/Cas9 mediated genome engineering can generate precise genetic modification or be used alongside homologous recombination to correct the genome of early human tripronuclear (3PN) zygotes and 2PN zygotes (Tang et al., 2017; Liang et al., 2015; Kang et al., 2016). These studies hold great promise for next-generation therapeutics and prospective parents who carry genetic mutations that lead to diseases, through preventing the transfer of these mutations to their offsprings.

Despite its therapeutic potential, the technical issues surrounding CRISPR technology hinder it from being clinically used. The first challenge includes off-target effects. Large genomes may consist of DNA sequences that are identical or closely resembling the target sequence, resulting in non-specific cleavage of Cas nuclease at non-target gene areas and giving rise to mutations. Depending on where the mutation is created, it may lead to cell death or transformation. Comparatively, off-target mutations are found more frequently in human cells than in mice and zebrafish (Hwang et al., 2013; Yang et al., 2013). Several papers have reported that these off-target mutations are heritable in humans as well, emphasising the need to improve CRISPR/Cas9 specificity before clinical application. As far as it is concerned, methods such as optimisation of sgRNA design, usage of paired nCas9s, paired dCas9-FokI nucleases, and enhanced Sp-Cas9 have been formulated to address this concern (Barrangou and Doudna, 2016; Hsu et al., 2014). Rapid advances have also been made to enhance the sensitivity of the techniques used for its evaluation throughout the whole genome. However, there remains the question of whether or not off-target effects are accountable for in a therapy that targets a single site within billions of DNA sequences, involving a large number of cells, and is designed specifically and differently among individual patients.

The second major challenge to the clinical application of gene editing is to enhance the efficiency of homology-directed recombination (HDR)-mediated precise gene modification while decreasing non-homologous end-joining (NHEJ)-induced indel production. As shown by various studies, the HDR-NHEJ ratio can be regulated by either changing the expression of machinery mediating DNA repair or optimising delivery methods and timing. A more desirable HDR-NHEJ ratio can be achieved through synchronisation of the cell cycle or utilisation of small molecules as well (Maeder and Gersbach, 2016). More recently, an endonuclease known as Cpf1 has also been shown to improve HDR. This variant not only allows the production of staggered cuts, but also needs shorter lengths of RNA (Zetsche et al., 2015).

Thirdly, there are difficulties with the delivery of Cas9 into tissues or cells to achieve therapeutic effects. The transfection of nuclease and gRNA expression-cassette bearing plasmid DNA is acknowledged as the prominent strategy to deliver nucleases into cells. Despite being straightforward, this procedure is not widely used in gene and cell therapy for various reasons, including low transfection efficiency of primary cells, likelihood that plasmid fragments are randomly inserted into the gene, cytotoxicity associated with the use of DNA, and bacterial DNA sequences present in plasmid backbones (Maeder and Gersbach, 2016; Wang et al., 2016). Adeno-associated virus (AAV)-based vectors are favored for use in somatic gene therapy as they induce a mild immune response, are not pathogenic, and are capable of targeting non-dividing cells. However, these vectors are limited by their packaging limit, in which the coding sequences of Sp-Cas9, the most well-used Cas9 and its sgRNA are almost reaching (Cox et al., 2015; Maeder and Gersbach, 2016). Consequently, the development of non-DNA-dependent alternatives including pre-assembled protein-RNA complexes paved the way for novel methods for delivery in gene therapy. For example, pure nuclease proteins or Cas9 protein-gRNA complexes are directly injected into cells. This has enabled successfully large quantities of genomic modification via electroporation, microinjections or lipid-mediated transfection. In comparison with vector-mediated nucleotide delivery methods, CRISPR-mediated genome targeting through pre-assembled protein-RNA complexes delivery methods results in improved fidelity as well as reduced cell toxicity, allowing the safety problems associated with the introduction of foreign DNA to be avoided (Maeder and Gersbach, 2016).

Finally, an issue that stems from genetic mosaicism, presumably as a result of nuclease activity succeeding the one-cell stage, still remains unresolved. Recently, Tu et al., reported that tagging Cas9 with ubiquitin-proteasomal degradation signals can facilitate the degradation of Cas9, reducing mosaic mutations and hence increases its ability to modify genomes in non-human primate embryos (Tu et al., 2017). Although problems arise from CRISPR-mediated gene editing, the technology is rapidly progressing with significant emphasis on pioneering and strategical enhancement.

With the advent of CRISPR/Cas9 technology, gene editing can be regularly and more efficiently performed in several species of organisms, ranging from insects and plants to rodents and primates. It can also be carried out in pluripotent stem cells and in human somatic cells for the purposes of basic research (Barrangou and Doudna, 2016; Baltimore et al., 2015). As CRISPR became increasingly used, research groups took genome editing a step further by inducing changes to the human germline in the hopes of correcting genetic diseases. This was first carried out in 2015 when Liang et al., demonstrated how these methods could be applied to human embryos by using CRISPR/Cas9 to cleave the endogenous beta-globing gene (HBB) off human tripronuclear zygotes, aiming to analyse the practicability and effectiveness of editing their genome to bring about therapeutic effects (Tang et al., 2017; Liang et al., 2015; Kang et al., 2016). Editing of the germline suggests that these modifications can be transmitted to successive generations, rendering it highly favorable over somatic cells if genes carrying familial disease-causing mutations could be successfully targeted. Not only will the modification of human embryo cells prevent the transmission of a known, inherited disease to the offspring from the prospective parents, it also alleviates the burden of carrying such a disease on the child (Savulescu et al., 2015).

While embryonic genomic editing has proven successful when carried out in animals, considerable technical problems must be attended to before it can be safely and predictably applied in humans as previously discussed. Referring to the study performed by Liang et al., there was a low efficiency of HDR of the endogenous β-globin gene (*HBB*), production of mosaic embryos, and formation of off-target mutations as shown through whole-exome sequencing and T7E1 assays (Liang et al., 2015). However, significant attention has been placed on off-target mutations especially in the context of human embryos. This is because editing of the embryo implicates that both advantageous gene corrections and detrimental effects like off-target mutations can be passed down to countless generations. As such, the use of CRISPR to genetically edit the human germline has sparked an ethical debate within the scientific community, questioning whether or not the benefits indeed outweigh the risks and raising the issue of informed consent. In this situation, informed consent of the offspring receiving the undesirable effects cannot be obtained, and there is uncertainty over who takes liability for genetic damage to be passed down several generations (Rodriguez, 2016).

Furthermore, another ethical concern arises from the risk of using CRISPR/Cas9 for non-therapeutic purposes such as enhancement. Preferred phenotypic characteristics could be achieved through genome editing of somatic or germ cells, going one step closer towards “designer babies”. For example, athletic performance or intellectual abilities may be improved, posing social problems if only specific individuals are able to receive this enhancement. Once again it also raises the issue of informed consent (Rodriguez, 2016). To prevent the abuse of CRISPR/Cas9 technology, clear regulations must be placed as guidance to draw the line between the ethical and socially acceptable use of gene editing and its misuse.

In light of addressing these issues, the International Summit on Human Gene Editing in Washington DC has been held to evaluate the technical concerns, and impacts of genome editing on the society and scientific research alike. Taking into account both the advantages and downsides of embryo gene editing research, the committee has concluded that such research will not be prohibited, although extreme caution must be exercised when utilising it. The use of germline editing in research trials can potentially be conducted, however prior to approval for clinical trials, further evaluation and progress must be made such that the benefits greatly outweigh the risks. Nonetheless, embryo gene modification must only be carried out under strict regulations (http://nationalacademies.org/gene-editing/consensus-study/index.htm). In this regard, we advocate for the application of both international guidelines and national policies to supervise and monitor research on human embryo gene editing. While common scientific and technical difficulties are encountered by the healthcare industry and academia, the differing cultural and economical backgrounds of each country subjects it to varying ethical issues. Therefore international guidelines are placed merely to answer consensus questions and as a basis so that individual countries legislate the appropriate, and specific respective oversight policies (Bosley et al., 2015).

Being relatively new, CRISPR/Cas9 technology has not yet been exploited to its maximum capabilities. However, it has already revolutionised genomic research, significantly affecting biomedical research and delivering the clinical advantages that previous treatments could not offer. In the future, use of CRISPR for genome editing could reduce the global burden of incurable diseases such as genetic disorders, cancer or HIV/AIDS, and potentially benefiting millions of people worldwide. Although the technology is predicted to advance further, various amendments must be made to make it more specific, safe, and efficient. There also remains the risk of improper use, raising ethical concerns that stir up doubt within the society. Aside from the regulation of use, other societal concerns include ensuring that future benefits outweigh the risks, policy decisions consider the values of the society and that the diverse perspective of individuals, countries, and cultures are respected. These factors affect the conditions and circumstances in which CRISPR/Cas9 will be used.

Prior to the publication of the above-mentioned papers on human embryonic genomic editing, commentaries have also been published by prestigious journals including Nature and Science, greatly discouraging this type of research and moratorium for ethical and legal reasons (Baltimore et al., 2015; Lanphier et al., 2015). While opinions within the scientific community are divided with some supporting, and others encouraging the prohibition of research on genome modification without providing convincing reasons, we strongly felt that a complete ban will not only hinder the development of future treatments, it is unfeasible as well, especially considering the high accessibility of CRISPR/Cas9 technology. The consequences of imposing such a ban remain questionable especially the impact on disabled people both in the present and future (Lanphier et al., 2015). Disabled people will not be protected by laws that differentiate on the grounds of disease labels. However, the views of disability groups and others that strive to protect the rights of these people and improve their social situation are often overlooked. Their opinions on the use of genetic approaches that intervene somatic and germ cells are neglected as well.

In summary, while genome editing using CRISPR-Cas9 allows for personalised medicine as well as the correction of human genetic diseases such as cystic fibrosis, several ethical considerations would need to be resolved prior to its clinical applications. The side effects of germline editing have often been cited as a justification for the cessation of its use on human germ cells. Arguments for embryonic gene modification supports its clinical use despite the suggested side effects due to the overall benefit that preventing the transmission of heritable genetic diseases brings. In addition, research on embryonic gene editing could also be expanded for non-therapeutic uses, provided that it is solely for research purposes. This is especially applicable for CRISPR-Cas9 and other germline editing technologies that may be used to resolve non-clinical scientific problems. In this regard, the US National Academies of Sciences, Engineering, and Medicine has recently made recommendations to maximize the benefits to human health of any applications of genome editing (http://nationalacademies.org/gene-editing/consensus-study/index.htm). While heritable germline editing is still underway, such clinical application is plausible in the foreseeable future. In fact, a recent paper by Hong et al. in 2017 proves that much improvement has already been made in the technology within the past 4 years. The paper reports the effective, specific, high HDR CRISPR/Cas9-mediated correction of the heterozygous Myosin-binding protein C (*MYBPC3*) pathogenic mutation involved in the pathogenesis of hypertrophic cardiomyopathy. Genome editing was carried out in human pre-implantation embryos, resulting in embryos without mosaicism and off-target mutations (Ma et al., 2017). More researches must be carried out to evaluate the reproducibility of the technique and before proceeding with its clinical application. Nonetheless, this proves that CRISPR/Cas9 has the ability to effectively and safely target heritable germline mutations to correct genetic diseases.

At the moment, it is certain that the CRISPR/Cas9 technology itself does not pose a threat, and the breakthroughs this technology can bring in therapeutics provides further support for its use in genetic editing of somatic cells. Moving forward, due to the rapid improvement of CRISPR/Cas9 technology, continual assessment and discussions will be necessary from time to time to ensure that this technology is utilised safely and responsibly.
